# Epidemiology of Glaucoma: The Past, Present, and Predictions for the Future

**DOI:** 10.7759/cureus.11686

**Published:** 2020-11-24

**Authors:** Karen Allison, Deepkumar Patel, Omobolanle Alabi

**Affiliations:** 1 Ophthalmology, Mount Sinai Hospital, New York, USA; 2 Public Health, New York Medical College, School of Health Science and Practice, Valhalla, USA; 3 Public Health, New York Medical College, Valhalla, USA

**Keywords:** primary open angle glaucoma, epidemiology and biostatistics, disease burden

## Abstract

Glaucoma is a multifactorial optic degenerative neuropathy characterized by the loss of retinal ganglion cells. It is a combination of vascular, genetic, anatomical, and immune factors. Glaucoma poses a significant public health concern as it is the second leading cause of blindness after cataracts, and this blindness is usually irreversible. It is estimated that 57.5 million people worldwide are affected by primary open-angle glaucoma (POAG). People over 60 years of age, family members of those already diagnosed with glaucoma, steroid users, diabetics, as well as those with high myopia, hypertension, central cornea thickness of <5 mm, and eye injury are at an increased risk of glaucoma. By 2020, it is expected that approximately 76 million people will suffer from glaucoma with that number estimated to reach 111.8 million by 2040.

In this article, we perform an extensive literature review focusing on the epidemiology of glaucoma and try to determine the number of people affected; we categorize them by sex, location, and level of income. Furthermore, we strive to estimate the future projection of the disease in the next 20 years (2040) while determining the disease burden, including the cost involved in treating and preventing the disease and the disease and disability projection of glaucoma.

## Introduction and background

Glaucoma is a group of progressive optic neuropathies characterized by a degeneration of retinal ganglion cells and retinal nerve fiber layers that result in changes in the optical nerve head [1]. Glaucoma is associated with intraocular pressure (IOP)-related damage to the optic nerve, which results in the loss of retinal ganglion cells [2].

Glaucoma is the leading cause of irreversible blindness worldwide and is associ­ated with a reduced quality of life [3]. Risk factors include age and frailty, gender, myopia, genetics, family history, smoking, race, systemic hypotension and hypertension, vasospasm, use of systemic or topical steroids, migraine, obstructive sleep apnea syndrome, and most significantly, increased IOP [4-6].

There are two major types of glaucoma: primary and secondary glau­coma. Both of these have two major subtypes (open-angle and angle-closure) according to the underlying anatomy and pathophysiology [1]. Primary or idiopathic glaucoma results from open-angle and closed-angle glaucoma with no identi­fiable cause while secondary glaucoma has an identifiable cause of increased IOP that causes optic nerve damage [1]. Open-angle glaucoma can be classified into primary open-angle glaucoma (POAG), normal-tension glaucoma (NTG), and secondary open-angle glaucoma. POAG is characterized by an increased IOP with pro­gression of the optic nerve; NTG is characterized by normal IOP with progression and optic neuropathy, and secondary open-angle glaucoma is characterized by elevated IOP and/or optic neuropathy [1].

Angle-closure glaucoma can be classified into primary angle-closure glaucoma (PACG) and secondary closed-angle glaucoma. PACG is further classified into acute (closure of anterior chamber angle with a sudden increase in IOP) and chronic (closure of the anterior chamber angle with a gradual increase in IOP or development of peripheral anterior synechiae) [1]. Secondary closed-angle glaucoma is the closure of the anterior chamber angle with increased IOP due to identifiable causes [1]. The incidence of blindness is higher in angle-closure glaucoma despite it being less common than open-angle glaucoma [1]. Glaucoma occurs at two time periods: early-onset exhibiting Mendelian inher­itance (<40 years) and adult-onset forms that are inherited as complex traits (>40 years) [7]. Mutations in the genes causing early-onset glaucoma are rare with many biological effects while variants contributing to adult-onset glaucoma are common with few biological effects [7]. 

NTG can occur as a result of rare mutations involving an optineurin (OPTN) missense mutation (E50K) and copy number variations (CNVs) involv­ing Tank-binding protein 1 (TBK1); mutations in these two genes account for 2-3% of NTG [7]. Juvenile open-angle glaucoma is a type of open-angle glaucoma that develops before the age of 40 years and can be the result of myocilin (MYOC) mutations [7]. Myocilin mutations account for 8-36% of juvenile open-angle glaucoma and 2-4% of adult-onset POAG [7]. Recent genome-wide association studies completed for POAG in European Caucasian and Asian populations have identified ABCA1, AFAP1, GMDS, PMM2, TGFBR3, FNDC3B, ARHGEF12, GAS7, FOXC1, ATXN2, and TXNRD2 as being significantly associated with glaucoma [7]. Other recent genome-wide association studies have identified PLEKHA7, COL11A1, PCMTD1-ST18, EPDR1, CHAT, GLIS3, FERMT2, and DPM2-FAM102 as being responsible for PACG in Asian populations [7].

**Figure 1:**
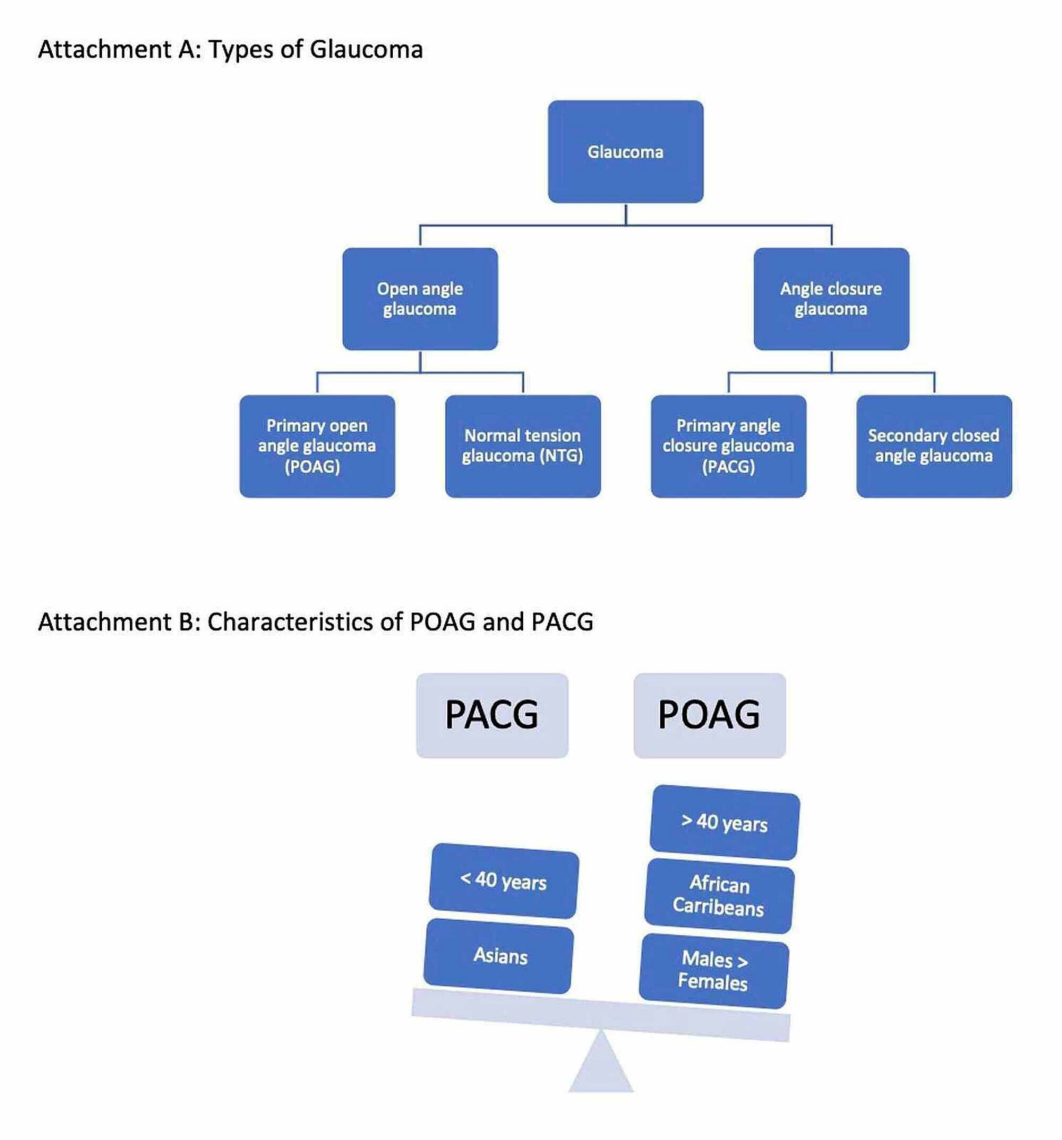
Types of Glaucoma and characteristics of POAG and PACG POAG: primary open-angle glaucoma; PACG: primary angle-closure glaucoma

The various types of glaucoma are depicted in Figure 1.

## Review

Prevalence of glaucoma

An estimated 57.5 million people worldwide are affected by POAG with a global prevalence of 2.2% [7]. In Europe, 7.8 million people were affected by POAG and the total prevalence is 2.51% [8-10]. The most common type of glaucoma in the UK is POAG, affecting 2% of individuals older than 40 years and 10% of individuals older than 75 years, particularly African-Caribbean people; PACG is not as prevalent and only affects 0.17% of individuals younger than 40 years, particularly East Asians [4]. One study has indicated that socioeconomic differences or inequalities have affected glaucoma services [7-9].

Regional/racial variations in the prevalence of glaucoma have been attributed to genetic and possible environmental differences. The susceptibility gene loci are significantly associated with POAG. Genes involved in the IOP regulation have been studied, and some African populations and the Igbo tribe of Nigeria, a rather homogenous ethnic group, have the highest prevalence of glaucoma [8-11]. Additionally, the high age-specific prevalence of glaucoma in Nigerian adults aged 40 years and above suggests that the severity of glaucoma begins at an earlier age and at a more aggressive course in blacks than in Caucasians and some Asians [11]. This could be a result of a lack of early diagnosis and poor access to treatment. Not surprisingly then, blacks develop POAG earlier than individuals of other ethnicities [2]. They are six-fold more likely to be affected by POAG than whites, while Mongolians and Burmese are more likely to be affected by PACG than POAG compared to whites [6,10]. The prevalence of glaucoma varies within each ethnicity depending on the country of birth [6,10].

Similarly, Kelly et al. [12] have estimated that among people aged 40 years and above, 2.2% of whites have glaucoma compared to 5.7% of blacks. Tham et al. [10] have predicted that the number of people aged 40-80 years who have glaucoma will increase from 64.3 million in 2013 to 76 million by 2020, and the total prevalence of POAG was 3.54% for individuals between the age of 40 and 80 years. The prevalence of glaucoma increases with age and can thus be associated with age-related diseases like macular degeneration, vascular diseases, and obstructive sleep apnea [6]. POAG is strongly correlated with age: its prevalence is highest among older Hispanic or Latino individuals (18%), black individuals (15%), white individuals (7%), and Asian individuals (5%) [6]. Finally, there are gender differences in glaucoma as well. There was a reported 36% higher prevalence of glaucoma in males than females [8,10]. Men are also noted to be at higher risk for POAG and women for PACG [6-10].

Kyari et al.'s [11] 2015 Nigeria Blindness Survey is so far the largest national population-based survey of eye disease in an ethnically diverse, indigenous black African population. They have described precise estimates of the prevalence of glaucoma in this region. The study sample was nationally representative by age, gender, ethnicity, rural/urban residence, and socioeconomic status with a high response rate. The results were generalizable to the whole country and people of the West African diaspora around the world [11]. The results indicated that 1.1-1.4 million Nigerian adults have glaucoma with the vast majority unaware of their disease status; one in every 20 Nigerian adults above 40 years have glaucoma and one in five of those individuals are blind [11].

There is a lower reported rate of glaucoma in African countries because surveys in African countries may have had a limited diagnostic capacity for glaucoma. However, Kyari et al. [11] have found that the prevalence of glaucoma in Nigeria is similar to that in Temba, South Africa and slightly higher than in South African Zulus and in Kongqa, Tanzania but lower than in Tema, Ghana, and Akinyele, Southwest Nigeria [11]. The surveys also show a pattern of higher prevalence of glaucoma in West Africa than in South Africa, which in turn is higher than the prevalence in East Africa [11]. The prevalence of glaucoma is lower in Nigeria than in Barbados and similar to black populations in the United States (US). It is slightly higher than in Asian populations and much higher than white populations in the US and Europe [11]. Furthermore, the prevalence of glaucoma is higher in Nigeria than in Brazil, Iran, indigenous populations in Australia, and Qatar [11].

Future projection of the disease

Another of Tham et al.'s [10] predictions include an increase in the number of people aged 40-80 years who have glaucoma from 76 million in 2020 to 111.8 million by 2040. Individuals from Europe, North America, and Oceania will contribute only a slight increase to the number of POAG and PACG cases from 2013 to 2040, and this 47.1% increase is mostly attributable to Asia and Africa. Individuals from Asia will be responsible for the increase of 18.8 million (79.8%) POAG cases and nine million (58.4%) PACG cases from 2013 to 2040; individuals from Africa will contribute a 10.9 million (130.8%) increase in glaucoma cases from 2013 to 2040 [10].

In the US, Kelly et al. [12] have estimated that there will be approximately 226,000 visually impaired people in the state of Georgia and about 100,000 of them would be blind by 2050. Based on a 47% increase in Georgia’s population by 2050, there will be about 254,047 cases of glaucoma among people aged 40 years and above [12].

Disease burden

Glaucoma is not easily detected and can thus go undiagnosed, thereby leading to an irreversible loss of vision. This is shocking when, according to the Vision & Eye Health Surveillance System (VEHSS) in 2017, there were 3,973,400 people with diagnosed glaucoma who had a Medicare (fee for service) treatment claim [13]. Of this number, 16,200 were between 18-39 years of age; 235,100 were between 40-64 years of age; 3,000,300 were between 65-84 years; and 721,900 were 85 years and older [13]. When classified according to gender, 2,395,600 of the diagnosed glaucoma cases were females and 1,577,800 were males [13]. In 2018, the crude annual prevalence of diagnosed glaucoma was 29.76% in black, non-Hispanics, 22.74% in Asians, 19.80% in Hispanics, 18.04% in North American Natives, and 17.50% in white, non-Hispanics [13].

Most glaucoma patients experience vision defects in tasks involving central and near vision (reading), mobility outside the home, difficulty in walking, stair-climbing, face recognition, and driving [14]. In addition, glaucoma is a significant predictor of depression after adjustment for demographic factors and multiple comorbidities with a prevalence estimated around 10% [14,15].

The cost of glaucoma includes direct, short-term costs to the patient or the healthcare system such as medications, office visits, diagnostic testing, and surgery as well as patient and societal costs in terms of loss of income/productivity and the long-term cost of vision loss [16]. The direct costs of glaucoma include medical bills, cost of medications, and transportation costs while indirect costs include low productivity at the workplace and days missed from work [17]. Furthermore, the burden of glaucoma extends to the families of patients, the healthcare system, and society at large [18-19].

What do these costs entail? Firstly, McGinley et al. [20] estimate that patients with glaucoma are more likely to be admitted to the hospital for a fall than those without glaucoma. The associated costs were estimated at £200,568 per year or £2,845 per patient. Furthermore, the mean number of bed days for falls in patients with glaucoma was 440, which in turn has a financial and operational impact on the hospital that needs the beds [19-20].

Other studies have found that glaucoma patients incurred an extra $2,903 annual total healthcare costs and $2,599 higher non-outpatient costs compared to individuals without glaucoma [16]. Prager et al. [16] found that Medicare beneficiaries with glaucoma were more likely to have inpatient hospitalizations, require home health aide visits, have a high mean total and non-outpatient costs, and worse ophthalmologic and non-ophthalmologic outcomes compared to their counterparts. Ultimately, the mean annual total costs were higher for patients with glaucoma ($16,760) than patients without glaucoma ($13,094); this remained higher even after the exclusion of outpatient costs ($14,273 vs. $11,024) [16].

Comparatively, the economic burden of glaucoma alone on the American economy is $2.9 billion [3]. The cost of treating and preventing glaucoma is about $5.8 billion per year in the US with the annual medical cost of glaucoma projected to be as high as $12 billion by 2032 and $17.3 billion by 2050 [18-19]. The annual direct cost of glaucoma treatment in the US ranges from $623 to $2,511 depending on its severity [21].

One reason for the high costs of glaucoma is the cost of medication. In the US, the annual cost of glaucoma topical medication ranged from $71.13 (timolol) to $1,548.26 (Allergan) with the average brand name costing $1,165.65 and the average generic medication costing $281.95 [21]. However, the annual cost of glaucoma topical medication ranged from $86.06 (timolol) to $514.48 (Cosopt - Merck) in Canada with the average brand name costing $306.76 and the average generic costing $143.44 [21].

All brand name medications were more expensive in the US than in Canada. For generic medications, six of 10 medications were 4.1 times more expensive in the US and the remaining four were 2.8 times more expensive in Canada [21]. From 2006 to 2013, the real prices of US brand name medications rose from 29% to 349%; in Canada, they rose from 9% to 16% [21]. Within that same time period, the real prices of US generic medications rose from -23% to 58% and from -38% to 0% in Canada [21].

In 2013, the US spent $329 billion on prescription drugs, and this amount is significantly higher than the per capita healthcare spending in Canada because prices are not regulated and are subject to market forces and insurance reimbursement policies [21]. Canadian-patented medication prices are regulated by the Patent Medicines Price Review Board (PMPRB) who ensures that prices are set at the median price from France, Germany, Italy, Sweden, Switzerland, the UK, and the US [21]. The PMPRB also ensures that new entry drug prices are set relative to the cost of the current therapies, and existing patented medications cannot increase more than the Consumer Price Index [21].

Access to glaucoma medications was significantly related to price and health insurance status. Most patients choose the least costly alternative. Others choose treatments based on more adverse effects for therapies with greater efficacy [19]. Most physicians are not aware of medications’ costs to patients and simply prescribe glaucoma medications based on their efficacy [19]. Additionally, patients felt that diagnostic testing accounted for one-third of glaucoma-related costs excluding the cost of medications [19].

Finally, there is the impact of poverty on glaucoma. The National Health Interview Survey found that people with a poverty income ratio of <1.5 were less likely to receive eye care or pupil dilation examinations in the past 12 months compared to people with a poverty income ratio of >5 [22]. Similarly, patient education was an important indicator for the adherence to topical glaucoma medication in glaucoma patients in Taiwan [23].

Advanced direction

The high prevalence and high rate of blindness make glaucoma a public health concern and a priority among healthcare planners and policymakers with an emphasis on the need for glaucoma care pathways for early detection and treatment to prevent blindness [11]. Detection at earlier stages is vital to prevent the progression of glaucoma [24].

One way of doing this is through teleglaucoma, which is the application of electronic technologies (through electronic transmission of high-resolution stereoscopic fundus photographs) to ophthalmic instruments to identify glaucoma cases and those at risk of glaucoma [24]. Teleglaucoma is particularly effective in rural areas. Several studies have shown that there is a moderate agreement between digital optic nerve assessments and slide films as well as a good correlation between cup-to-disc ratios from teleglaucoma and ophthalmoscopy [24].

There is a direct benefit to patients with a cost savings of approximately $2,474.60. There is only a mean cost of $9,22.77 for every patient screened and $1,098.67 for every detected case [24]. Verma et al. (2014) also found that the majority of teleglaucoma patients did not require an in-person consultation and could be managed with teleglaucoma [25]. Teleglaucoma is effective at screening negative cases and the technology offers poor quality images in only 10.4% of images. It has improved access to ophthalmologists and had a referral rate of 12.5% to the ophthalmologist [24]. Early detection approaches in teleglaucoma have successfully reduced the probability of patients in a blind state by 24% and maintained 13% more patients at mild-stage glaucoma in comparison to in-person care [24-25].

In Alberta, teleglaucoma was found to have an incremental cost-effectiveness ratio (ICER) of about $27,460 per quality-adjusted life-year (QALY) (cost per patient served) relative to in-person examination [24]. The World Health Organization provides the threshold for cost-effective interventions (the ICER associated with the implementation of the intervention is less than the country’s GDP); in Alberta’s population, teleglaucoma is cost-effective because it has an ICER below Alberta’s GDP [24]. Conclusively, teleglaucoma provides opportunities for collaboration among the primary care provider, the optometrist, and the ophthalmologist to optimize patient health outcomes [24-25].

In addition to teleglaucoma, nationwide implementation of screening middle-aged African Americans can decrease the rate of undiagnosed glaucoma from 50% to 27%; this would enable patients to more effectively leverage current treatment options to reduce the risk of bilateral blindness later in life [26]. However, treatment efficacy and efficiency appear to differ between racial/ethnic groups within controlled clinical trials. Long-term treatment of African American and Caucasian patients with pharmacologically uncontrolled glaucoma showed that African Americans experienced better visual outcomes when argon laser trabeculoplasty (ALT) was performed prior to a two-step trabeculectomy procedure compared to Caucasians who experienced better visual outcomes when ALT was performed between the two rounds of trabeculectomy [26].

**Figure 2:**
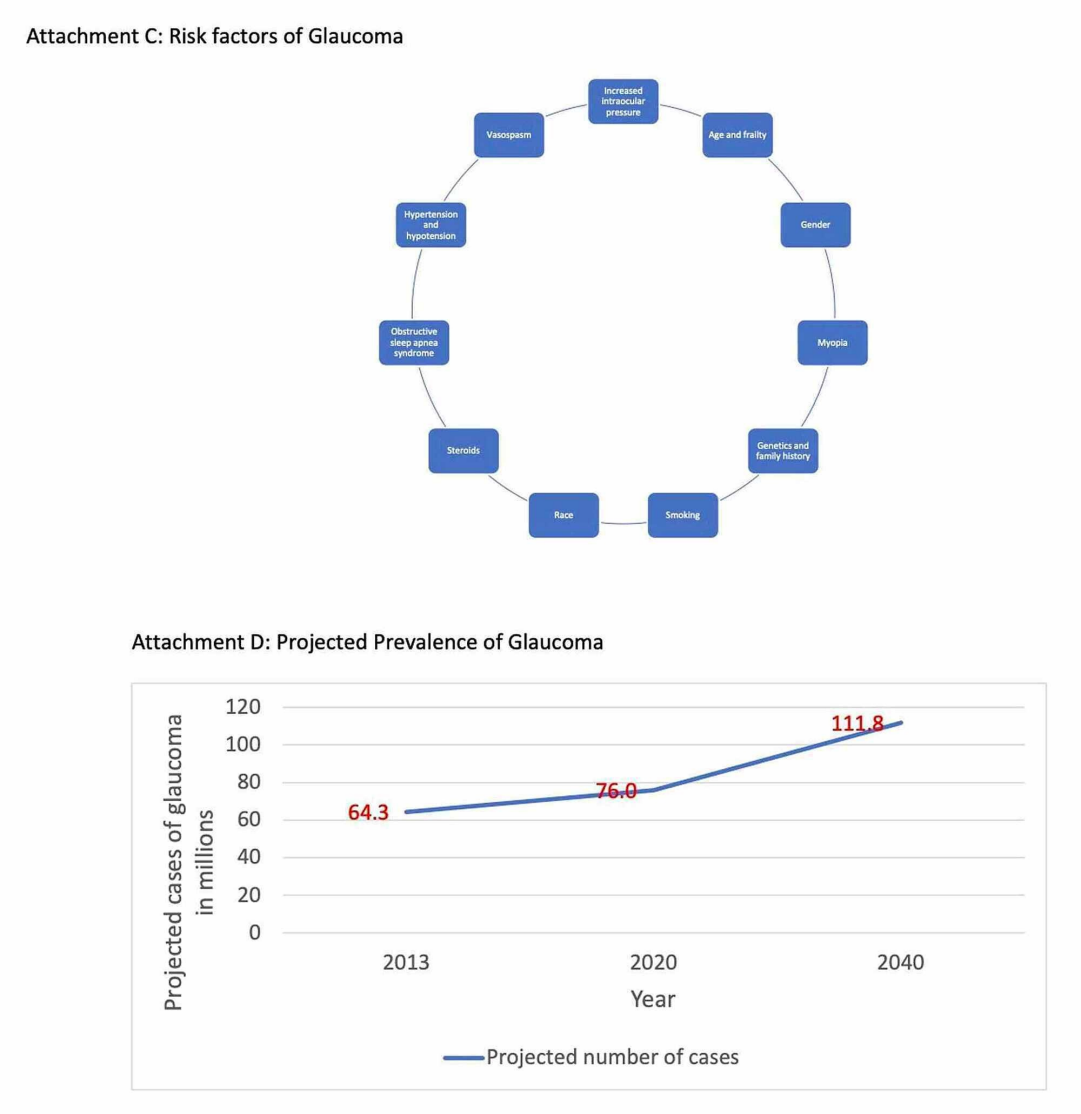
Risk factors and projected prevalence of glaucoma

**Figure 3:**
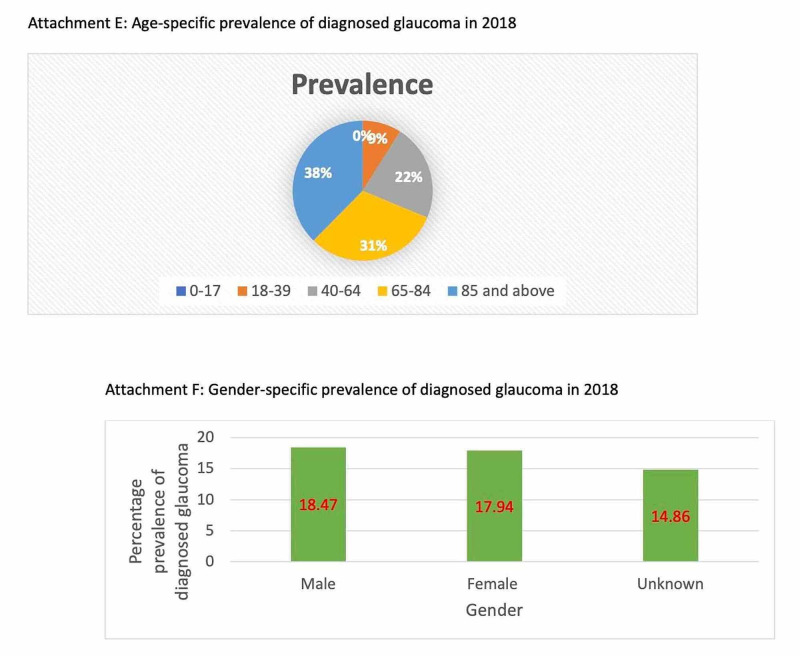
Age-specific and gender-specific prevalence of diagnosed glaucoma in 2018

**Figure 4:**
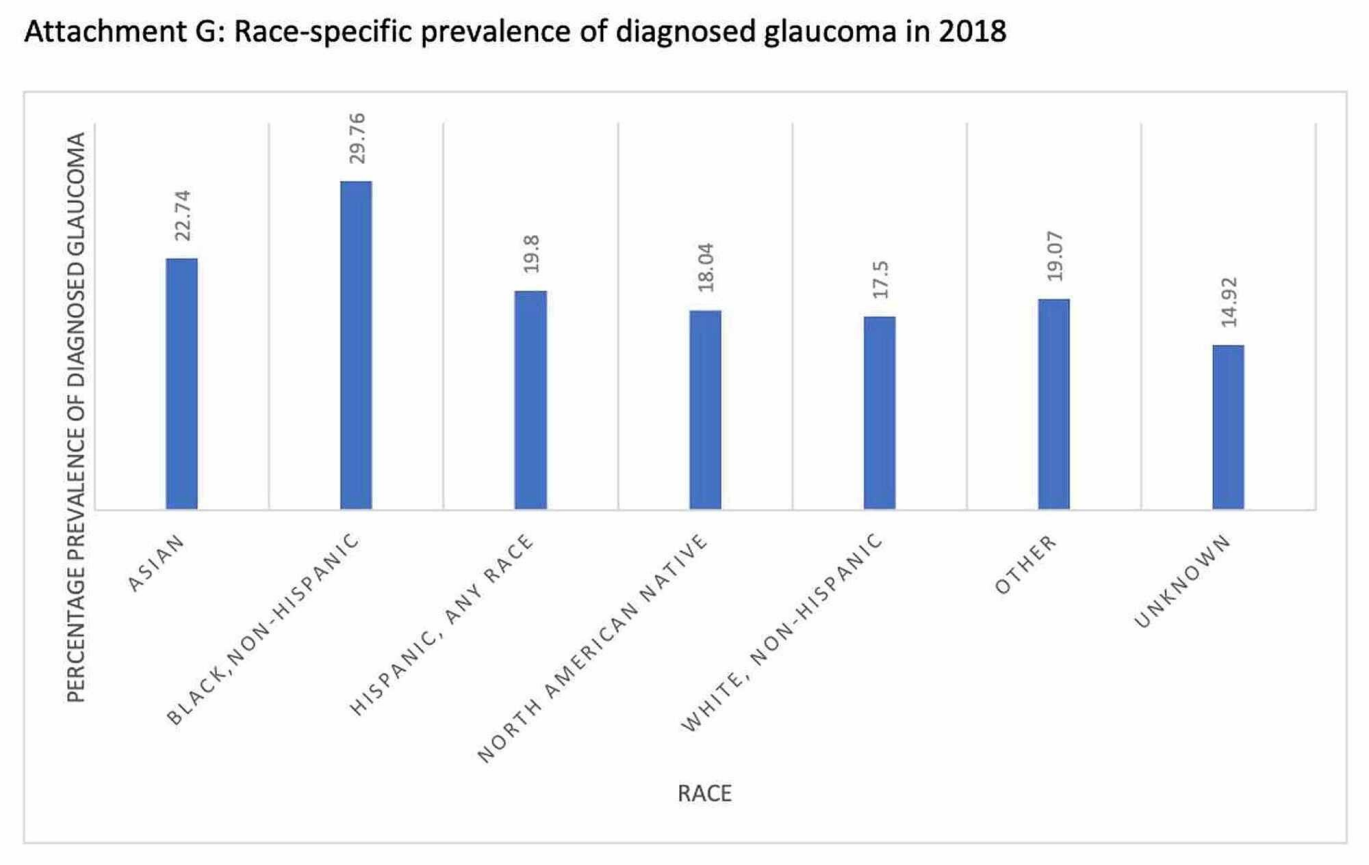
Race-specific prevalence of diagnosed glaucoma in 2018

**Figure 5:**
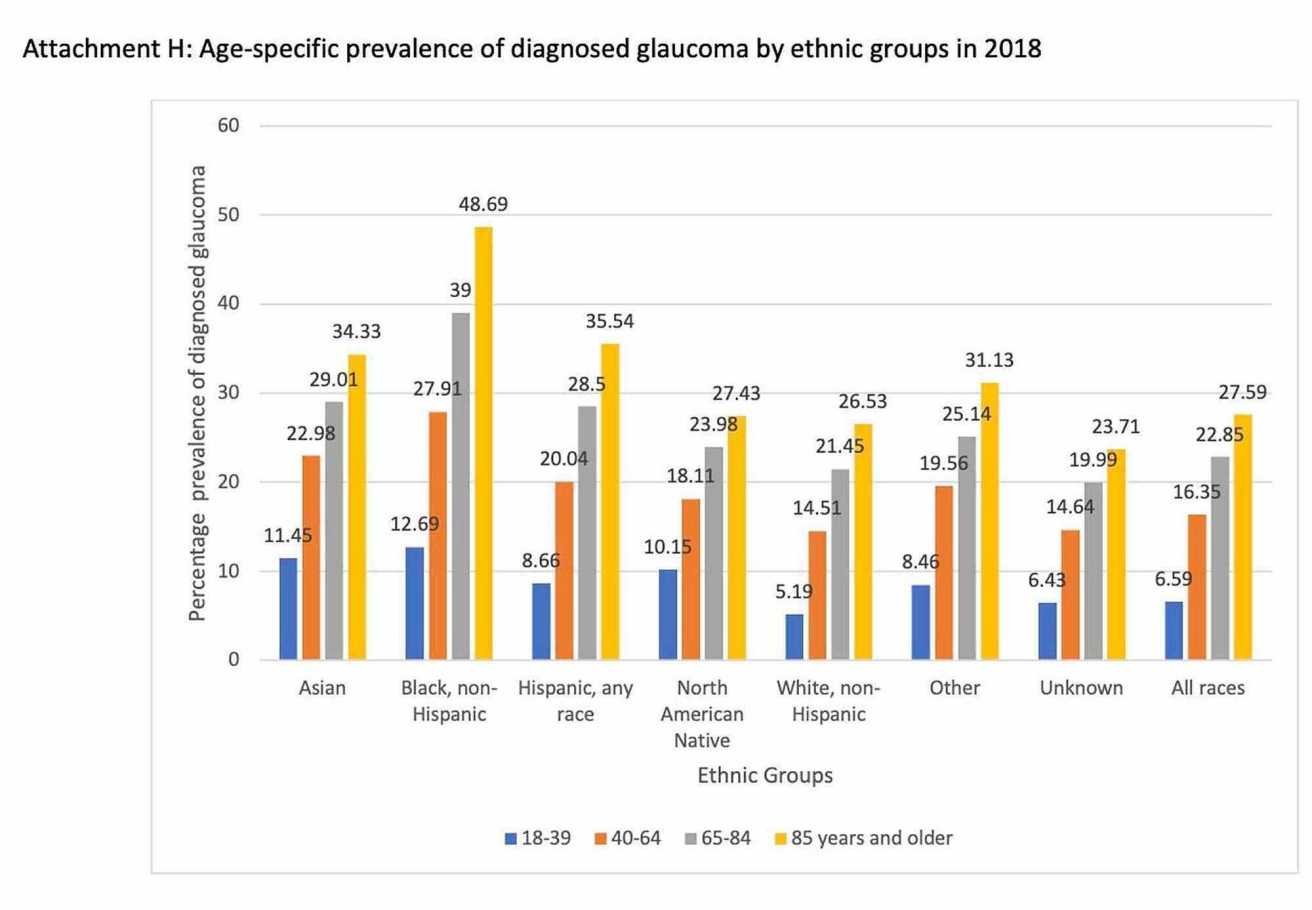
Age-specific prevalence of diagnosed glaucoma by ethnic groups in 2018

**Figure 6:**
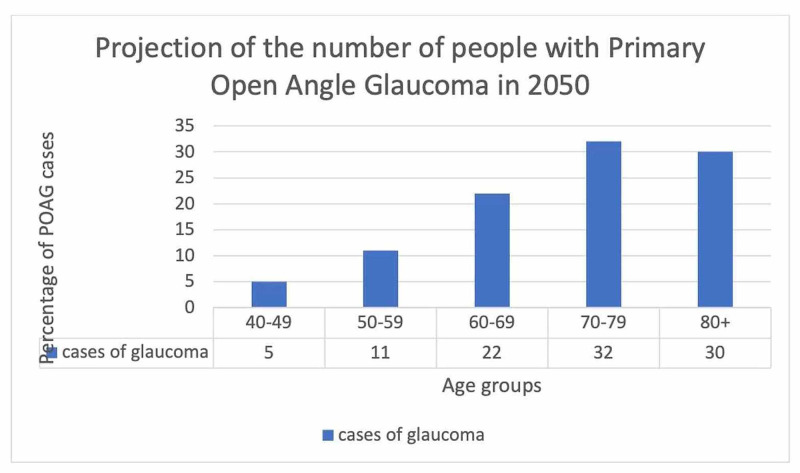
Future projections of glaucoma POAG: primary open-angle glaucoma

The risk factors associated with glaucoma and the prevalence of the condition based on various parameters such as age, gender, race, ethnic groups are depicted in Figures 2, 3, 4, 5. Figure 6 illustrates the future projections related to glaucoma.

## Conclusions

Based on our study, glaucoma represents a significant healthcare burden both in the US and worldwide. Changes need to be made to reduce the incidence of glaucoma and stop it from being a bigger public health threat. Some recommendations include implementing telehealth in glaucoma, regular glaucoma screenings, stronger education, more medications/surgery studies, and diversity in healthcare providers. Due to the high inheritance rate of glaucoma, people with a family history of glaucoma should be educated about it and screened for it as early as possible. Likewise, people with a high-risk factor for glaucoma and Africans should undergo genetic counseling to see if they test positive for glaucoma genes.
